# Tumeurs sub-mandibulaires: profils épidémiologiques et histologiques

**DOI:** 10.11604/pamj.2014.18.64.2102

**Published:** 2014-05-18

**Authors:** Pegbessou Plaodezina Essobozou, Ndiaye Malick, Diom Evelyne, Thiam Amadou, Diouf Mame Sanou, Boube Djafarou, Ndiaye Cire, Tall Abdourhamane, Diallo Bay Karim, Ndiaye Issa Cheikh, Diouf Raymond, Diop Malick

**Affiliations:** 1 Clinique Universitaire ORL et Chirurgie Cervicofaciale, Centre Hospitalier Universitaire de Fann de Dakar, Sénégal

**Keywords:** Glande submandibulaire, tumeur, epidémiologie, histologie, submandibular gland, tumor, epidemiology, histology

## Abstract

**Introduction:**

Il s'agit de déterminer les profils épidémiologiques et histologiques des tumeurs submandibulaires.

**Méthodes:**

Il s'agissait d'une étude rétrospective et descriptive de 10 ans (1er janvier 2000 au 31 décembre 2009), réalisée dans le service universitaire d'ORL de l'hôpital de Fann. Etaient inclus dans cette étude tous patients porteurs d'une tumeur submandibulaire (opéré ou non), confirmée par un document histologique.

**Résultats:**

Vingt-une tumeurs submandibulaires ont été colligées. L’âge moyen des patients était de 34,42 ans (± 14,10), avec des extrêmes de 2 et 55 ans. Quinze patients (71,4%) étaient de sexe féminin, soit un sex-ratio de 0,4. Les résultats histologiques étaient obtenus à partir de 4 biopsies et de 17 pièces opératoires. Dans 13 cas (61,9%) la tumeur était bénigne et dans 8 cas (38,1%) la tumeur était maligne. L'adénome pléomorphe dans 12 cas (57,1%), le carcinome épidermoïde dans 4 cas (19%) et l'adénocarcinome dans 2 cas (9,5%) étaient les types histologiques fréquents. Treize (13) patients étaient porteurs d'une tumeur bénigne, dont huit (8) patients étaient de sexe féminin. Huit (8) patients étaient porteurs d'une tumeur maligne. Sept patients étaient de sexe féminin.

**Conclusion:**

Cette étude sur les tumeurs submandibulaires est marquée par une prédominance féminine et une fréquence élevée des adénomes pléomorphes et des carcinomes épidermoïdes.

## Introduction

La glande submandibulaire fait partie des glandes salivaires principales. Ces glandes salivaires principales (parotide, submandibulaire, sublinguale) sont le siège de plusieurs pathologies: inflammatoires et infectieuses, lithiasiques, tumorales, malformatives. La pathologie tumorale des glandes salivaires fait environ 3% de toutes les tumeurs du corps et 6% des tumeurs de la tête et du cou [1]. Les tumeurs parotidiennes constituent la majorité des tumeurs salivaires et l'adénome pléomorphe est le type histologique fréquemment décrit [2, 3]. Au niveau de la glande submandibulaire la pathologie tumorale serait de 5-15% de l'ensemble des tumeurs salivaires [4–7] mais la proportion des tumeurs malignes serait plus importante que celles des tumeurs parotidiennes [8]. Cette étude sur les tumeurs submandibulaires a pour objectif de déterminer les profils épidémiologiques et histologiques.

## Méthodes

Il s'agissait d'une étude rétrospective et descriptive de 10 ans (1er janvier 2000 au 31 décembre 2009), réalisée dans le service universitaire d'ORL de l'hôpital de Fann. Etaient inclus dans cette étude tous patients porteurs d'une tumeur submandibulaire (opéré ou non), confirmée par un document histologique. L'histologie a été obtenue à partir des pièces opératoires et des prélèvements biopsiques. La pièce opératoire a été obtenue après une submandibulectomie par voie externe. La biopsie a été faite sur des tumeurs inopérables. Les patients dont l'ablation de la glande submandibulaire a été faite: au cours d'un évidement ganglionnaire pour un cancer de voies aérodigestives supérieures, pour une submandibulite lithiasique ou allergique n’étaient pas inclus dans cette étude. Les données ont été collectées sur une fiche d'enquête à partir des dossiers des malades et des registres d'histologie.

## Résultats

**Figure 1:**
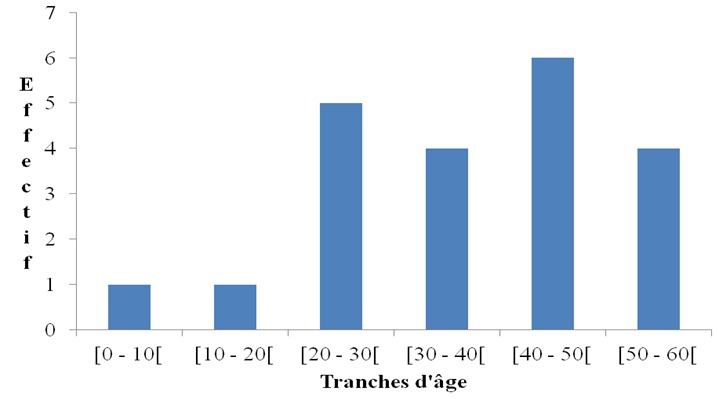
Répartition des patients suivant les tranches d’âge

Au cours de la période d’étude, 21 tumeurs submandibulaires ont été colligées. L’âge moyen des patients était de 34,42 ans (± 14,10), avec des extrêmes de 2 et 55 ans. Le nombre de tumeurs submandibulaires était plus important dans la tranche d’âge de 40 à 50 ans. La Figure 1 représente la répartition des patients suivant les tranches d’âge. Quinze patients (71,4%) étaient de sexe féminin et 6 patients étaient de sexe masculin (29,6%). Le sex-ratio était de 0,4. Les résultats histologiques étaient obtenus à partir de 4 biopsies et de 17 pièces opératoires. Dans 13 cas (61,9%) la tumeur était bénigne et dans 8 cas (38,1%) la tumeur était maligne. L'adénome pléomorphe dans 12 cas (57,1%) et le carcinome épidermoïde dans 4 cas (19%) étaient les types histologiques fréquents. Le Tableau 1 représente les différentes entités histologiques retrouvées. Treize (13) patients étaient porteurs d'une tumeur bénigne, dont huit (8) patients étaient de sexe féminin, soit un sex ratio de 0,625. L’âge moyen de ces patients était de 34,23 ans avec des extrêmes de 16 et 50 ans. Huit (8) patients étaient porteurs d'une tumeur maligne. Sept patients étaient de sexe féminin, soit un sex-ratio de 0,14. L’âge moyen était de 34,75 ans avec des extrêmes de 2 et 55 ans. Le Tableau 2 est la représentation des tumeurs submandibulaires selon le sexe.

**Tableau 1:** Répartition des différentes entités histologiques

	Effectif	Pourcentage (%)
**Tumeurs bénignes**		
Adénome pléomorphe	12	57,1
Fibrome	1	4,8
**Tumeurs malignes**		
Carcinome épidermoïde	4	19
Adénocarcinome	2	9,5
Cylindrome	1	4,8
Tératome malin	2	4,8
**Total**	**21**	**100**

**Tableau 2:** Répartition des tumeurs suivant le sexe

	Féminin	Masculin
**Tumeurs bénignes**		
Adénome pléomorphe	7	5
Fibrome	1	0
**Tumeurs malignes**		
Carcinome épidermoïde	4	0
Adénocarcinome	2	0
Carcinome adénoïde kystique	1	0
Tératome malin	0	1
**Total**	15	6

## Discussion

Notre étude a porté sur les tumeurs submandibulaires. Il est difficile de retrouver dans la littérature des études portant simultanément sur les tumeurs malignes et bénignes de la glande submandibulaire. La plupart des études porte sur: les glandes salivaires, les submandibulectomies [9, 10], les tumeurs bénignes submandibulaires [11], tumeurs malignes submandibulaires [12–14] et des cas cliniques [15–17]. L’âge moyen de découverte des tumeurs submandibulaires était de 34,42 ans dans notre étude; ce résultat est proche de celui de Moatemri et al [1], qui était de 40 ans. Mais cet âge était différent de celui de Ethunandan et al [14] et de Rapidis et al [10] (54 ans et 60 ans respectivement). La prédominance féminine rapportée par certains auteurs [10, 14] a été confirmée dans notre étude. D'autres auteurs ont trouvé une prédominance masculine [1, 9]. L'adénome pléomorphe reste la tumeur bénigne la plus fréquente des tumeurs submandibulaires et de toutes les tumeurs salivaires [16–18]. Elle se présente comme une tuméfaction à croissance lente, indolore, ferme ou dure, régulière ou irrégulière. Elle prédomine chez le sujet de sexe féminin, son taux de récidive est de 5-30% [17]. La dégénérescence maligne des récidives serait de 25% [17]. Dans le groupe des tumeurs malignes, le carcinome adénoïde kystique [19] est le type histologique souvent rencontré [13, 20–22]. Elle est une tumeur asymptomatique; rarement on note la douleur voire un engourdissement de la langue [16]. Le tabagisme et l'alcoolisme sont retrouvés dans les antécédents des patients. Elle a un tropisme nerveux élevé à l'origine des récurrences et des métastases pulmonaires [23, 24]. Ce résultat est contraire au résultat de notre étude qui rétrouve une prédominance de carcinome épidermoïde. Le carcinome épidermoïde est rarement décrit dans les tumeurs des glandes salivaires, mais sa fréquence est reconnue dans les tumeurs des voies aérodigestives. Pour certains auteurs, les carcinomes épidermoïdes des glandes salivaires sont considérés comme des métastases des cancers cutanés (carcinomes et mélanomes), ou de carcinomes épidermoïdes des VADS. Ceci conduit à rechercher systématiquement ces lésions cutanées ou des VADS, voire d'autres lésions de transfert. Ces métastases représenteraient 5% de l'ensemble des tumeurs des glandes salivaires. Ces métastases seraient dues une diffusion par contiguité d'une tumeur non salivaire à une glande salivaire ou par diffusion hématologique [25]. Dans cette étude, les patients n'avaient pas de lésion cutanée, ni de lésion des VADS.

## Conclusion

Cette étude sur les tumeurs submandibulaires est marquée par une prédominance féminine et une fréquence élevée d'adénomes pléomorphes et de carcinomes épidermoïdes.
